# Association of Galectin-3 and Soluble ST2, and Their Changes, with Echocardiographic Parameters and Development of Heart Failure after ST-Segment Elevation Myocardial Infarction

**DOI:** 10.1155/2019/9529053

**Published:** 2019-10-10

**Authors:** Agata Tymińska, Agnieszka Kapłon-Cieślicka, Krzysztof Ozierański, Monika Budnik, Anna Wancerz, Piotr Sypień, Michał Peller, Paweł Balsam, Grzegorz Opolski, Krzysztof J. Filipiak

**Affiliations:** 1st Department of Cardiology, Medical University of Warsaw, Warsaw, Poland

## Abstract

**Purpose:**

To investigate the association of galectin-3 (Gal-3) and soluble ST2 (sST2) and their follow-up changes with the development of heart failure (HF) and echocardiographic parameters of HF (ejection fraction, atrial and ventricular size, left ventricular hypertrophy, e′, and E/e′) in patients with ST-segment elevation myocardial infarction (STEMI) treated with primary percutaneous coronary intervention (pPCI).

**Methods:**

A prospective, observational study, BIOSTRAT (Biomarkers for Risk Stratification After STEMI), enrolled 117 patients between October 2014 and April 2017. Gal-3 and sST2 serum collection and echocardiography were performed twice (during index hospitalization and on a control visit at one-year follow-up). The primary endpoint was HF onset at one-year follow-up. Secondary assessments included associations of biomarker concentration with echocardiographic indices of systolic and diastolic dysfunction at baseline and at one year.

**Results:**

Mean baseline concentrations of Gal-3 and sST2 (7.5 and 26.4 ng/mL, respectively) were significantly increased at one-year follow-up (8.5 ng/mL and *p* < 0.001 and 31.4 ng/mL and *p* = 0.001, respectively). Patients who reached the primary endpoint (50 patients (48%)) had significantly higher baseline concentrations of both biomarkers and a higher Gal-3 level at one year compared to patients who did not. Both Gal-3 and sST2 were predictors of the primary endpoint in univariate logistic regression analysis, but only Gal-3 remained significant in multivariate analysis. There was no clear association between both biomarkers and echocardiographic parameters.

**Conclusions:**

Baseline, but not one-year, changes of Gal-3 and sST2 concentrations may be useful for risk stratification after STEMI. However, only Gal-3 was the independent predictor of HF development at one-year observation. This trial is registered with NCT03735719.

## 1. Introduction

Acute myocardial infarction (AMI) initiates left ventricular remodeling (LVR) and may lead to the development of heart failure (HF) [1]. Accessible diagnostic tools commonly used in HF such as natriuretic peptides and New York Heart Association (NYHA) classification reflect already overt clinical HF [2, 3]. Troponin and creatine kinase reflect myocardial damage, but their usefulness in predicting long-term LVR is limited [3]. Recent guidelines on HF management stressed that HF onset may be delayed or prevented through certain interventions, such as pharmacotherapy, postinfarction rehabilitation, or modification of HF risk factors [3]. Therefore, it is important to identify potential markers, which would be more informative of HF preclinical stages to recognize patients with an increased risk of HF onset, and to start treatment in advance.

Numerous studies suggest that, in addition to natriuretic peptides, circulating galectin-3 (Gal-3) and soluble interleukin-1 receptor-like 1 (sST2) are independent markers of adverse outcomes in HF [4–10]. These two biomarkers have been already recommended for an additive risk stratification in the American guidelines for the management of HF [11]. These biomarkers represent pathophysiological pathways other than cardiac troponins or natriuretic peptides. Gal-3 participates in inflammation and profibrotic pathways, while sST2 is a biomarker of inflammation, cardiac mechanical strain, and tissue fibrosis, both of which may predict LVR [12, 13]. Both biomarkers are involved in many regulatory processes and might be useful in estimation of the risk of adverse cardiac remodeling and development of HF. Few small clinical studies have recently suggested a potential role of baseline Gal-3 and sST2 concentrations in predicting adverse outcomes after AMI [14–17], but there is insufficient data on the clinical usefulness of measurements of both biomarkers one year after AMI.

The aim of this study was to investigate the association of Gal-3 and sST2 concentrations and their changes at one-year follow-up with the development of clinically overt HF and echocardiographic indices of HF (left ventricular (LV) ejection fraction (LVEF), atrial and ventricular size, LV hypertrophy based on LV mass index (LVMI), diastolic tissue velocities at the mitral annulus (e′), and E/e′ ratio) in patients after ST-segment elevation myocardial infarction (STEMI) treated with primary percutaneous coronary intervention (pPCI).

## 2. Methods

### 2.1. Study Design and Population

**Figure 1:**
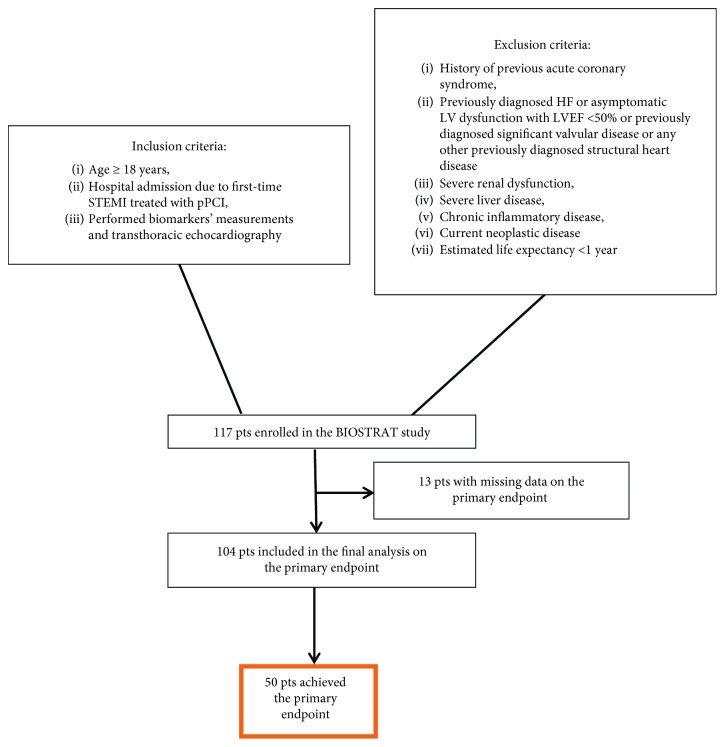
Flow chart of patient enrolment in the current analysis. HF: heart failure; pPCI: primary percutaneous coronary intervention; STEMI: ST-segment elevation myocardial infarction.

BIOSTRAT (Biomarkers for Risk Stratification After STEMI) was a prospective, observational, single-centre study, conducted between October 2014 and April 2017. The study included 117 consecutive patients admitted due to first-time STEMI treated by pPCI. STEMI was diagnosed based on standard algorithms [18]. Participants who agreed to sign informed consent were included based on the following main criteria: (1) age ≥ 18 years and (2) hospital admission due to first-time STEMI treated with pPCI. The main exclusion criteria were (1) history of previous acute coronary syndrome, (2) previously diagnosed HF or asymptomatic left ventricular (LV) dysfunction with LVEF < 50% or previously diagnosed significant valvular disease or any other previously diagnosed structural heart disease, (3) severe renal dysfunction (plasma creatinine level > 220 mmol/L (≈2.5 mg/dL) and/or creatinine clearance < 30 mL/min), (4) severe liver disease, (5) chronic inflammatory disease, (6) current neoplastic disease, and (7) estimated life expectancy < 1 year.  Figure 1 shows patient enrolment in the current study.

The study protocol was approved by the local ethics committee and registered at ClinicalTrials.gov (NCT03735719).

### 2.2. Data Collection and Gal-3 and sST2 Measurements

Data on baseline clinical characteristics, clinical examination, results of diagnostic tests performed during index hospitalization (ECG, echocardiography, coronary angiography, and biochemistry [including maximum concentrations of cardiac troponin I (cTnI), creatine kinase myocardial band (CK-MB), N-terminal pro-B-type natriuretic peptide (NT-proBNP), and high-sensitivity C-reactive protein (hs-CRP)]), and pharmacotherapy were collected.

Transthoracic echocardiography was performed within 48 hours of hospital admission in the Department's Echocardiography Laboratory using Philips EPIQ 7 Ultrasound Machines (Philips Medical Systems, Andover, Massachusetts, USA) by 3 certified echocardiographers (second degree accreditation in echocardiography of the Section of Echocardiography of the Polish Cardiac Society). LVEF was assessed by biplane Simpson's modified rule [19]. Other assessed parameters included atrial and ventricular size, LV posterior wall and septal thickness, LV hypertrophy based on LVMI: LVMI > 95 g/m^2^ for female and LVMI > 115 g/m^2^ for male, mitral inflow velocities, and early diastolic tissue velocities at the lateral and medial mitral annulus (E′ lat and E′ med). All measurements were performed according to the recommendations of the American Society of Echocardiography and the European Association of Cardiovascular Imaging [19, 20].

Blood samples for Gal-3 and sST2 were collected 72-96 hours after hospital admission. Separation of plasma was performed 1 hour after blood collection by centrifugation at 3500 rpm for 15 min at ambient temperature. Then, specimens were stored at –80°C until analyzed after trial completion. Serum concentrations of Gal-3 were assessed using a Human Galectin-3 Quantikine ELISA Kit® (BIOKOM, Janki, Poland) and of sST2 with a Presage ST2 Assay (Genloxa Sp. z o.o., Puck, Poland). Calibration and standardization of these assays were performed according to the manufacturers' protocols.

At the one-year follow-up, a control visit in our department was conducted. Clinical examination, echocardiography, and collection of blood samples for biomarkers' measurements were performed.

### 2.3. Study Endpoints

The primary endpoint of our study was new HF onset at one-year follow-up. New-onset HF was defined as LVEF below 40% or HF-related hospitalization or ambulatory diagnosis of HF.

Secondary assessments included associations of the baseline and follow-up biomarker concentrations with echocardiographic indices of systolic and diastolic dysfunction at baseline and at one year.

### 2.4. Statistical Analysis

On the basis of literature data [21] regarding Gal-3 concentration, the sample size of the study group was calculated. In the de Boer et al. study, the median concentration of Gal-3 in the entire study group (*n* = 247) was 13.4 (11.4-16.2 ng/mL; SD: 3.6). The study endpoint was LVEF < 50% after four months of observation since PCI performed in AMI. Patients who reached the study endpoint had higher median Gal-3 concentration 14.8 (12.5-18.2 ng/mL; SD: 4.2) comparing to those who had not (median 13.0 (11.2-15.4 ng/mL; SD: 3.1)). Therefore, theoretically (considering normal distribution and assuming 10% loss to follow-up) to reach statistical significance (with power of 80%), 114 patients should be included in the study.

Normally distributed continuous variables were presented as mean values and standard deviations, while ordinal variables and nonnormally distributed continuous variables, as median values and interquartile ranges (IQR). Categorical data were presented as numbers and percentages. Changes in concentrations of biomarkers were also calculated as the one-year level minus the baseline level and correlated with clinical variables. Significance of differences between groups was determined by Fisher's exact test for categorical variables and a Mann-Whitney *U* test for continuous and ordinal variables, respectively. The Wilcoxon signed-rank test was used to compare repeated measurement of biomarkers. The logistic regression model was performed to identify predictors of the primary endpoint. A *p* value of ≤0.05 was considered significant for all tests. In order to maintain an adequate events per predictor variable (EPV) value, we did not include all variables that were significant in the univariate analysis to the Cox proportional hazards regression model [22]. All tests were two-tailed. Pearson's and Spearman's correlation coefficients were used for parametric and nonparametric variables, respectively. Youden's J statistic was performed to determine the optimal biomarker's cut-off point for prediction of the primary endpoint. Receiver operating characteristic (ROC) curves were plotted for Gal-3 and sST2 in relation to the primary endpoint. Statistical analyses were performed using SPSS software, version 22 (IBM SPSS Statistics 22, New York, USA).

## 3. Results

### 3.1. Baseline Characteristics and One-Year Follow-Up

The final analysis included 104 patients who had completed the one-year follow-up. In all cases, the ischemia-related artery was revascularized using drug-eluting stents (*n* = 109) or balloon angioplasty alone (*n* = 5) during PCI. In most of the cases, the ischemia-related artery was the right coronary artery (*n* = 49) and the left anterior descending (*n* = 47), while less frequently was observed the stenotic circumflex artery (*n* = 14), diagonal artery (*n* = 2), obtuse marginal artery (*n* = 2), posterior descending artery (*n* = 2), and intermediate artery (*n* = 1). A total of 96 (92%) patients had echocardiography performed, and 89 (86%) patients had blood samples collected at the control visit at one year. Fifty out of 104 patients (48%) reached the primary endpoint.  Table 1 presents clinical, biochemical, and echocardiographic characteristics of patients who reached and who did not reach the primary endpoint. During the follow-up, 4 patients died and 13 patients had HF-related hospitalizations (6 patients had hospitalization for HF worsening; 8 patients had cardioverter defibrillator implanted). Causes of death included HF in 2 patients, MI in 1 patient, and a noncardiovascular cause in 1 patient.

**Figure 2:**
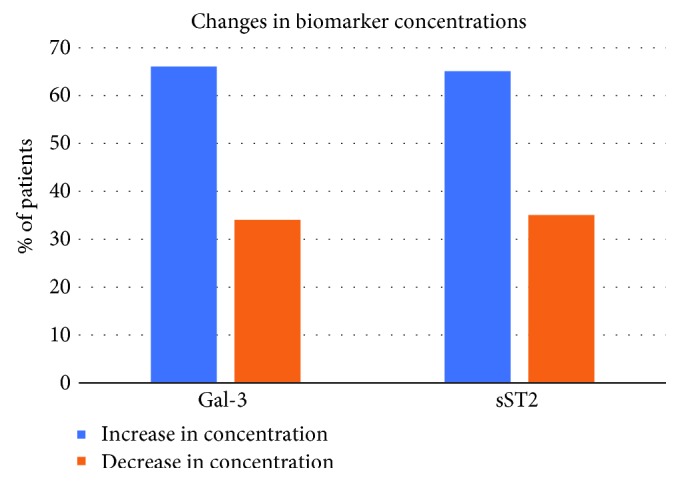
Changes^∗^ in biomarker concentrations. ^∗^Changes were calculated as the one-year level minus the baseline level of each biomarker.

At baseline, the mean value of Gal-3 was 7.5 (2.0-19.3) ng/mL and sST2 was 26.4 (6.1-89.9) ng/mL, while at one year their concentrations significantly increased to 8.5 ng/mL (2.5-19.7, *p* < 0.001) and 31.4 ng/mL (13.0-57.2; *p* = 0.001), respectively. Gal-3 and sST2 levels increased over time in a majority of patients (66% for Gal-3 and 65% for sST2) (Figure 2). Compared to patients who did not reach the primary endpoint, patients who developed HF at one year had significantly higher baseline concentrations of both biomarkers and a higher Gal-3 level at one year. However, there were no significant differences in sST2 levels at one year or in changes of both biomarkers during follow-up between the two groups (Table 1).

Additionally, patients in the highest quartiles of Gal-3 and sST2 concentration at baseline were more likely to develop HF during follow-up than patients in lower quartiles. A similar association was observed for the highest quartile of Gal-3 measured at one year (Table 2).

In ROC analysis, the area under the curve (AUC) for Gal-3 and sST2 (for prediction of the primary endpoint) was 0.61 and 0.50. Gal-3 concentration of ≥8.74 ng/mL had a sensitivity of 38%, a specificity of 81%, a negative predictive value of 58%, and a positive predictive value of 65% for prediction of the primary endpoint at follow-up (Youden's index). sST2 concentration of ≥34.48 ng/mL had a sensitivity of 27%, a specificity of 83%, a negative predictive value of 55%, and a positive predictive value of 60% for prediction of the primary endpoint at follow-up (Youden's index).

Both Gal-3 and sST2 were predictors of the primary endpoint in univariate logistic regression analysis, but only Gal-3 remained significant in multivariate analysis (Table 3).

### 3.2. Association of Gal-3 and sST2 with Echocardiographic Parameters

We correlated Gal-3 and sST2 concentrations at baseline and after one year and their changes with echocardiographic parameters ([Supplementary-material supplementary-material-1]). Correlation analysis revealed that higher baseline Gal-3 concentrations correlated inversely only with LV end-diastolic volume (LVEDV) at one year. There were no other significant correlations of baseline, follow-up, nor changes in Gal-3 concentration with echocardiographic parameters. Baseline sST2 values correlated positively with LV end-diastolic diameter (LVEDD), LV end-systolic volume (LVESV), and LV mass index (LVMI) and inversely with LVEF at one-year, but not with baseline echocardiographic parameters. Changes in sST2 concentration correlated positively only with LVEF at one year. There were no significant correlations of sST2 follow-up concentrations with echocardiographic parameters.

We also assessed echocardiographic parameters at follow-up by quartiles of baseline Gal-3 and sST2 concentrations, which is summarized in Table 2. Only participants with a higher sST2 level had lower LVEF at baseline and after one year, and patients with higher concentrations of both Gal-3 and sST2 at baseline were more likely to have LV hypertrophy initially and after one year. There was no clear association of rising quartiles with other echocardiographic parameters.

### 3.3. Correlation of Gal-3 and sST2 with Clinical Parameters

We performed a correlation analysis of Gal-3 and sST2 concentrations assessed at baseline and at one year and their changes with clinical parameters ([Supplementary-material supplementary-material-1]). A significant positive correlation was found between Gal-3 and sST2 in regard to baseline, but not to follow-up, nor to changes in biomarker concentrations. Baseline Gal-3 and sST2, changes in sST2 concentration, and follow-up levels of Gal-3 correlated positively with NT-proBNP. Baseline and follow-up levels of Gal-3 and sST2 correlated negatively with the glomerular filtration rate. Baseline and follow-up levels of Gal-3 and sST2 positively correlated and changes in sST2 concentration negatively correlated with a longer stay in the intensive cardiac care unit. Baseline Gal-3 and follow-up levels of sST2 correlated positively with the Killip class, and baseline sST2 positively correlated with NYHA at one year. Only baseline and follow-up Gal-3 correlated positively with age. Only changes in sST2 concentration correlated negatively with cTnI.

### 3.4. Comparison of Patients with Preserved and Reduced Ejection Fraction at One Year

Patients with LVEF < 50% at one year (30% of patients) had higher baseline concentrations of NT-proBNP, cTnI, CK-MB, and hs-CRP. There were no significant differences between both LVEF groups in terms of baseline, follow-up, nor changes in Gal-3 and sST2 concentrations (Table 4).

## 4. Discussion

The main finding of our study is that baseline Gal-3 and sST2 presented potential clinical utility in predicting HF development at one year among patients admitted primarily due to STEMI treated with pPCI. In the total STEMI population, concentrations of both biomarkers increased in most of the patients and were significantly higher after one year compared to the baseline concentrations. Patients with new-onset HF had significantly higher baseline levels of Gal-3 and sST2 compared to patients without developed HF. Importantly, Gal-3, but not sST2, was a predictor of the primary endpoint in the multivariate analysis.

MI provokes the inflammatory response with migration of a multitude of cells into the myocardium which initiates structural and biochemical changes in the infarcted and noninfarcted areas leading to LVR. Pathological processes such as myocardial fibrosis, hypertrophy, and changes of ventricular size result in the impairment of LV diastolic and systolic function [1, 23]. Natriuretic peptides are considered as well-proven diagnostic and prognostic biomarkers in HF, but they are not always reliable. Concentrations of natriuretic peptides may be increased in various clinical situations and may also stay low until advanced stages of the disease [11, 24, 25]. LVR may continue for weeks or months; therefore, there is a need for biomarkers that can reflect myocardial fibrosis and early identify patients at risk of HF, which may have important implications for postdischarge follow-up. Considering the contribution of inflammation and fibrosis in the progression of HF, the clinical utility of Gal-3 and sST2 for prediction of new-onset HF is of interest.

Gal-3 is mainly located in the myocardial extracellular matrix and cardiac fibroblasts, whereupon myocardial stress induces cardiac remodeling. In experimental data, Sanchez-Mas et al. observed that Gal-3 increases in the myocardium after MI with the maximum concentration achieved in the infarcted area during the first week, with a gradual decrease in the following weeks [26]. Interestingly, Gal-3 also increased in noninfarcted areas, and it seems that the increase in concentrations of Gal-3 in the early phase after MI contributes to the activation of repair functions in the damaged zone in order to maintain the geometry and function of the heart [26]. However, in the long term, chronic activation leads to tissue fibrosis and accelerates adverse LVR [26].

In an experimental study, sST2 concentrations increased steadily after MI with a maximum expression at 12–18 h [13]. Both Gal-3 and sST2 provide robust prognostic information in patients with existing HF in predicting an increased risk of cardiovascular mortality and events [27–30].

A few previous studies have shown the clinical utility of a single measurement of Gal-3 and sST2 in the diagnosis of HF after acute coronary syndrome [14–17, 31–33]. In our study, patients who reached the primary endpoint had increased baseline concentrations of Gal-3 and sST2. However, in the multivariate logistic regression analysis, only Gal-3 persisted an independent predictor of the primary endpoint. Jenkins et al. concluded that high sST2 concentrations in patients after MI were also associated with an increased risk of HF development independently of other prognostic factors such as age, sex, comorbidities, Killip class, and troponin T in 5 years of observation, so the follow-up period was longer than in our study.

Moreover, previously, it was proven that serial measurements of Gal-3 and sST2 provide additional prognostic information. van der Velde et al. observed in large cohorts of patients with acute and chronic HF that repeated measurements of Gal-3 provide more accurate prognostic information when compared to a single measurement. Authors showed that >15% increase of Gal-3 concentrations between measurements was associated with a 50% higher risk for subsequent HF morbidity and mortality, independently of age, sex, diabetes mellitus, left ventricular ejection fraction, renal function, HF medication, and NT-proBNP (*p* = 0.001) [28]. In the TRIUMPH (Translational Initiative on Unique and novel strategies for Management of Patients with Heart failure) study, investigators showed that serial sST2 measurements were a strong predictor of all-cause mortality and HF rehospitalization in patients with acute HF, independently of NT-proBNP [29]. Moreover, based on the results of the PREVEND (Prevention of Renal and Vascular Endstage Disease) study, conducted among the general population, the researchers suggested that the presence of elevated concentration of Gal-3 may be a predictor of the development of HF. However, this relationship was observed only in patients with increased baseline cardiovascular risk [34]. In addition, serial biomarker measurements have been shown to provide more accurate prognostic information compared to a single Gal-3 measurement [34]. The same was observed by Ghorbani et al. that traditional cardiovascular risk factors (older age, hypertension, diabetes, and BMI) were associated with a rise in Gal-3 levels over time and the largest changes in Gal-3 were in regard to the development of chronic kidney disease, HF, and all-cause mortality [35].

We demonstrated that both biomarkers' concentrations increased during one-year observation in the total population after STEMI, regardless of the occurrence of HF. However, there was no significant difference in sST2 concentrations between the two cohorts after one year. In contrast, van der Velde et al. observed that in patients after AMI Gal-3 concentrations increased, while sST2 levels decreased. However, the authors observed patients for only the first 4 months, when it can be expected that changes in biomarkers' concentrations may be more pronounced. They also observed higher baseline concentrations of the biomarkers, but they recruited subjects regardless of the type of acute coronary syndrome [21]. Accordingly, Sabatine et al. observed that baseline levels rather than subsequent values of sST2 appeared to be more predictive of cardiovascular death or HF. However, what one would expect is that subsequent values of NT-proBNP appeared to be more predictive than the initial value [36].

We observed that baseline but not follow-up sST2 concentrations significantly correlated with lower LVEF at follow-up. However, we also observed that the patients with increasing concentrations of sST2 had better LVEF than patients with decreasing sST2. This may reflect a greater mass of the vital myocardium and the reparative role of sST2 post-MI to prevent LV dilatation and preserve LVEF. A similar finding was observed by van der Velde in relation to Gal-3; however, in our study we did not observe a correlation between changes in Gal-3 concentration and LVEF.

The occurrence of HF with preserved ejection fraction (HFpEF) has risen significantly over the past decade and is characterized by the presence of diastolic dysfunction [3, 37–39]. Considering the contribution of Gal-3 and sST2 in inflammation and fibrosis and their significance in the progression of HF, we analyzed whether both biomarkers might be predictors of LVR and diastolic dysfunction. Our study showed an association between Gal-3 and sST2 and LV hypertrophy, but these biomarkers were not related to higher LV filling pressures (E/e′ ratio) and diastolic tissue velocities at the mitral annulus (E′).

### 4.1. Limitations

The study was mainly limited due to a relatively small number of patients, with approximately 10% lost to follow-up. In addition, some patients did not have a follow-up echocardiogram or did not have all parameters measured. Moreover, there are missing data on biomarker levels, including Gal-3 and sST2 at the control visit, and other biomarkers such as NT-proBNP from hospital stay.

## 5. Conclusions

These data highlight the potential role of Gal-3 and sST2 measurements after STEMI in the prediction of HF onset. However, baseline measurements of Gal-3 and sST2 showed greater clinical significance than values obtained after one year or changes in biomarker concentrations. In patients who achieved the primary endpoint, concentrations of both biomarkers were initially higher and Gal-3 was the predictor of the primary endpoint. In the entire study population, the biomarker levels were higher after one year. Both biomarkers were not associated with parameters of diastolic dysfunction; thus, it is unclear whether these biomarkers may be helpful in diagnosing and predicting HFpEF. There is a need for further studies to determine the predictive value and clinical utility of repeated measurements of sST2 and Gal-3 concentrations in patients after AMI.

## Figures and Tables

**Table 1:** Baseline characteristics of patients with and without new HF onset at one-year follow-up.

Variable	*n* = 104		*p* value
	Patients without HF at 1 year (*n* = 54)	Patients with HF at 1 year (*n* = 50)	
*Baseline characteristics*			
Age (years)	58.0 (53.0-67.3)	64.0 (57.0-70.8)	**0.04**
Male gender	72.2%; 39/54	70.0%; 35/50	0.83
BMI (kg/m^2^)	27.6 (24.0-29.7); *n* = 51	29.7 (25.2-33.2); *n* = 42	**0.04**
Moderate valve disease	1.9%; 1/54	8.0%; 4/50	0.19
Hypertension	59.3%; 32/54	64.0%; 32/50	0.69
Atrial fibrillation	0.0%; 0/54	10.0%; 5/50	**0.02**
Diabetes	13.0%; 7/54	30.0%; 15/50	0.053
Chronic kidney disease	14.8%; 8/54	22.0%; 11/50	0.45
COPD	5.6%; 3/54	6.0%; 3/50	1.00
Prior stroke or TIA	1.9%; 1/54	8.0%; 4/50	0.19
Peripheral artery disease	1.9%; 1/54	12.0%; 6/50	0.053
Current or former smoking	70.4%; 38/54	72.0%; 36/50	1.00

*Clinical status at hospital admission*			
Heart rate (b.p.m.)	75.0 (69.5-80.0)	80.0 (73.5-90.0)	**0.01**
SBP (mmHg)	130.0 (120.0-140.0)	130.0 (115.8-146.0)	0.49
DBP (mmHg)	70.0 (68.3-85.0)	80.0 (70.0-90.0)	0.16
Killip class	1 (1-1)	1 (1-2)	0.06
Intravenous diuretics during hospitalization	20.4%; 11/54	42.0%; 21/50	**0.02**
Extent of CAD, *n* (%)			
1-vessel	57.4%; 31/54	48.0%; 24/50	0.43
2-vessel	25.9%; 14/54	36.0%; 18/50	0.29
3-vessel	16.7%; 9/54	16.0%; 8/50	1.00

*Laboratory findings at admission*			
Hemoglobin (g/dL)	14.2 (13.5-15.1)	14.3 (13.6-15.7)	0.52
Serum creatinine (mg/dL)	0.96 (0.86-1.1)	0.9 (0.8-1.1)	0.69
eGFR (mL/min/1.73m^2^)	89.2 (65.2-111.7)	89.1 (60.0-119.2)	0.95
Serum sodium (mmol/L)	140.0 (138.5-141.7)	139.6 (137.7-142.0)	0.64
Serum potassium (mmol/L)	3.9 (3.5-4.2)	4.0 (3.7-4.2)	0.31
Total cholesterol (mg/dL)	188.0 (161.5-230.5); *n* = 52	182.5 (148.8-211.5); *n* = 48	0.36
LDL (mg/dL)	120.0 (76.8-154.5); *n* = 48	108.0 (82.0-133.0); *n* = 43	0.50
HDL (mg/dL)	43.5 (36.0-51.8); *n* = 52	45.0 (32.0-55.0); *n* = 47	0.39
Triglycerides (mg/dL)	135.0 (94.0-170.0); *n* = 51	134.0 (86.0-200.0); *n* = 47	0.75
hs-CRP peak (mg/dL)	2.8 (1.1-7.1); *n* = 51	4.2 (2.1-10.1); *n* = 48	**0.03**
Troponin I peak (ng/L)	16.0 (1.6-65.1); *n* = 51	46.7 (9.4-111.5); *n* = 49	**0.03**
CK-MB peak (U/L)	45.2 (5.3-184.1); *n* = 53	91.9 (32.8-212.9)	0.07
NT-proBNP peak (pg/mL)	514.0 (192.0-1479.8); *n* = 38	1917.0 (850.5-4258.8); *n* = 36	**0.001**
Gal-3 (ng/mL)	6.9 (4.6-8.0)	7.8 (6.5-10.0)	**0.002**
sST2 (ng/mL)	23.4 (17.0-29.9)	25.7 (20.1-34.5)	**0.04**

*Echocardiography*			
LVEF (%)	51 (45-55)	43 (35-49)	**<0.001**
LVEDD (mm)	4.8 (4.5-5.1)	5.0 (4.6-5.3); *n* = 49	0.30
LVEDV (mL)	100.0 (80.0-121.0); *n* = 33	106.0 (72.0-131.0); *n* = 39	0.80
LVESV (mL)	47.0 (38.0-60.0); *n* = 33	61.0 (38.0-86.0); *n* = 39	0.17
LVH^∗^	18.0%; 9/50	55.0%; 22/40	**<0.001**
LA dimension (mm)	3.8 (3.5-4.1)	3.9 (3.6-4.2); *n* = 49	0.15
E′ med (cm/s)	6.9 (6.0-7.8); *n* = 33	5.7 (4.2-7.7); *n* = 33	**0.03**
E/E′ med	11.0 (9.9-12.3); *n* = 36	12.4 (10.1-15.5); *n* = 36	0.06
E′ lat (cm/s)	8.9 (6.7-10.5); *n* = 33	6.2 (5.1-9.8); *n* = 32	**0.03**
E/E′ lat	8.4 (6.7-10.8); *n* = 36	10.0 (7.9-12.9); *n* = 35	0.08
TAPSE (mm)	21.0 (20.0-25.0); *n* = 53	22.0 (20.0-25.0)	0.85
Moderate mitral regurgitation	5.6%; 3/54	24.0%; 12/50	**0.01**

*Clinical status and laboratory findings at discharge*			
Heart rate (b.p.m.)	71.5 (63.0-80.0)	72.0 (67.5-83.0); *n* = 49	**0.002**
SBP (mmHg)	120.0 (110.0-131.0)	120.0 (110.0-136.0); *n* = 49	0.98
DBP (mmHg)	71.5 (63.0-80.00)	75.0 (65.5-80.00); *n* = 49	0.58
Hemoglobin (g/dL)	13.9 (12.8-14.6); *n* = 49	13.5 (12.3-14.7); *n* = 45	0.22
Serum creatinine (mg/dL)	1.0 (0.8-1.1); *n* = 47	0.9 (0.8-1.1); *n* = 45	0.78
eGFR (mL/min/1.73m^2^)	91.4 (76.2-112.5); *n* = 47	86.1 (60.1-114.2); *n* = 45	0.30
Serum sodium (mmol/L)	141.8 (140.1-142.9); *n* = 47	140.7 (138.8-143.1); *n* = 45	0.21
Serum potassium (mmol/L)	4.3 (4.1-4.6); *n* = 47	4.5 (4.2-4.7); *n* = 45	0.21

*Pharmacotherapy at hospital discharge* ^∗∗^			
ASA	100%; 54/54	100%; 49/49	1.00
Clopidogrel	94.4%; 51/54	83.7%; 41/49	0.11
Ticagrelor	5.6%; 3/54	16.3%; 8/49	0.11
Anticoagulant	3.7%; 2/54	14.3%; 7/49	0.08
Loop diuretic	5.6%; 3/54	46.9%; 23/49	**<0.001**
ACE-I	96.3%; 52/54	95.9%; 47/49	1.00
ARB	3.7%; 2/54	10.2%; 5/49	0.25
*β*-Blocker	90.7%; 49/54	95.9%; 47/49	0.44
Aldosterone antagonist	22.2%; 12/54	40.8%; 20/49	0.06
Ivabradine	0.0%; 0/54	4.1%; 2/49	0.22
Statin	98.1%; 53/54	95.9%; 47/49	0.60

*Biomarkers at follow-up*			
Gal-3 (ng/mL)	7.4 (5.3-9.5); *n* = 46	9.1 (7.4-11.2); *n* = 43	**0.01**
Increase in Gal-3 level from baseline	60.9%; 28/46	72.1%; 31/43	0.37
Change^∗∗∗^ in Gal-3 (ng/mL)	0.5 (-0.7-2.0); *n* = 46	1.7 (-0.3-3.1); *n* = 43	0.17
sST2 (ng/mL)	28.5 (24.2-33.3); *n* = 46	33.1 (25.7-40.0); *n* = 43	0.11
Increase in sST2 level from baseline	63.0%; 29/46	67.4%; 29/43	0.82
Change^∗∗∗^ in sST2 (ng/mL)	4.6 (-3.5-11.2); *n* = 46	5.2 (-4.2-13.7); *n* = 43	0.72

Bold values indicate *p* values < 0.05. ^∗^LVH was based on left ventricular mass index (LVMI): LVMI > 95 g/m^2^ for female, LVMI > 115 g/m^2^ for male. ^∗∗^In patients who survived to hospital discharge. ^∗∗∗∗^Changes in biomarker concentrations were calculated as the one-year level minus the baseline level of each biomarker. ACE-I: angiotensin-converting-enzyme inhibitor; ARB: angiotensin II receptor blocker; ASA: acetylsalicylic acid; b.p.m.: beats per minute; BMI: body mass index; CK-MB: creatine kinase-muscle/brain; COPD: chronic obstructive pulmonary disease; DBP: diastolic blood pressure; E′ lat: lateral early diastolic mitral annular velocity; E/E′ lat: early mitral inflow divided by lateral early diastolic mitral annular velocity; E′ med: medial early diastolic mitral annular velocity; E/E′ med: early mitral inflow divided by medial early diastolic mitral annular velocity; eGFR: estimated glomerular filtration rate; Gal-3: galectin-3; HDL: high-density lipoprotein; HF: heart failure; hs-CRP: high-sensitivity C-reactive protein; LA: left atrium; LDL: low-density lipoprotein; LVEDD: left ventricular end-diastolic diameter; LVEDV: left ventricular end-diastolic volume; LVESV: left ventricular end-systolic volume; LVH: left ventricular hypertrophy; LVEF: left ventricular ejection fraction; *n*: number; NT-proBNP: N-terminal pro-B-type natriuretic peptide; SBP: systolic blood pressure; sST2: soluble interleukin-1 receptor-like 1; TAPSE: tricuspid annular plane systolic excursion; TIA: transient ischemic attack.

**(a):** 

Variable	Baseline Gal-3				*p* value^∗^	Follow-up Gal-3				*p* value^∗^
	Quartiles					Quartiles				
	1	2	3	4		1	2	3	4	
*Primary endpoint*										
New-onset HF at follow-up	0.0%; 0/2	39.1%; 18/46	43.3%; 13/30	73.1%; 19/26	**0.01**	27.3%; 6/22	50.0%; 11/22	52.2%; 12/23	63.6%; 14/22	**0.02**

*Baseline echocardiography*										
LVEF (%)	44.5 (40.5-59.5); *n* = 6	48.0 (43.0-53.8); *n* = 52	50.0 (38.3-55.0); *n* = 30	45.0 (34.0-50.5); *n* = 29	0.09	49.0 (40.8-56.5); *n* = 22	49.0 (38.0-52.0); *n* = 22	48.0 (43.0-51.0); *n* = 23	45.0 (35.3-51.3); *n* = 22	0.26

*Follow-up echocardiography*										
LVEF (%)	45.0 (45.0-45.0); *n* = 1	56.0 (49.0-58.0); *n* = 44	56.0 (45.0-60.8); *n* = 28	53.0 (44.0-55.0); *n* = 23	0.25	56.0 (51.0-60.3); *n* = 22	54.0 (46.3-56.5); *n* = 22	55.0 (46.0-58.0); *n* = 23	55.0 (40.0-60.0); *n* = 22	0.58
LVEDV (mL)	142.0 (142.0-142.0); *n* = 1	108.5 (92.5-129.0); *n* = 38	105.0 (81.3-126.5); *n* = 22	84.0 (75.5-120.0); *n* = 16	0.33	115.5 (91.3-138.0); *n* = 16	114.0 (94.0-133.8); *n* = 18	101.0 (80.0-117.0); *n* = 19	87.0 (77.0-116.0); *n* = 18	0.22
LVESV (mL)	78.0 (78.0-78.0); *n* = 1	48.5 (40.0-62.0); *n* = 38	42.5 (31.5-61.5); *n* = 22	40.5 (33.0-66.5); *n* = 16	0.76	50.5 (35.5-73.5); *n* = 16	54.5 (38.0-77.0); *n* = 18	45.0 (30.0-62.0); *n* = 19	39.5 (32.8-63.0); *n* = 18	0.70
LVH^∗^	0.0%; 0/1	35.0%; 14/40	44.0%; 11/25	64.7%; 11/17	**0.04**	28.6%; 6/21	47.4%; 9/19	47.4%; 9/19	47.1%; 8/17	0.23
E/E′ med	12.0 (12.0-12.0); *n* = 1	10.7 (8.6-12.6); *n* = 42	9.0 (8.0-11.9); *n* = 27	10.0 (8.2-15.3); *n* = 22	0.54	8.5 (10.0-11.9); *n* = 20	10.8 (9.2-13.4); *n* = 22	9.7 (8.3-12.7); *n* = 23	9.4 (7.9-12.6); *n* = 22	0.80
E/E′ lat	8.1 (8.1-8.1); *n* = 1	8.0 (7.0-10.3); *n* = 42	8.5 (6.2-11.4); *n* = 27	8.8 (6.7-10.9); *n* = 22	0.97	7.3 (6.9-9.6); *n* = 20	9.0 (7.0-12.0); *n* = 22	8.8 (6.5-11.5); *n* = 23	8.4 (6.7-10.4); *n* = 22	0.44

**(b):** 

Variable	Baseline sST2				*p* value^∗^	Follow-up sST2				*p* value^∗^
	Quartiles					Quartiles				
	1	2	3	4		1	2	3	4	
*Primary endpoint*										
New-onset HF at follow-up	22.7%; 5/22	64.0%; 16/25	44.4%; 20/45	75.0%; 9/12	**0.004**	42.9%; 9/21	34.8%; 8/23	52.2%; 12/23	63.6%; 14/22	0.06

*Baseline echocardiography*										
LVEF (%)	50.0 (41.0-54.5); *n* = 29	47.0 (41.5-52.0); *n* = 29	48.5 (42.0-52.3); *n* = 46	44.0 (32.0-46.0); *n* = 13	**0.04**	49.0 (40.5-55.0); *n* = 21	48.0 (39.0-52.0); *n* = 23	48.0 (41.0-54.0); *n* = 23	46.5 (43.0-50.0); *n* = 22	0.75

*Follow-up echocardiography*										
LVEF (%)	56.0 (45.0-60.0); *n* = 18	56.0 (46.8-56.0); *n* = 24	55.0 (51.0-58.0); *n* = 43	48.0 (35.0-55.0); *n* = 11	**0.047**	56.0 (44.5-58.0); *n* = 21	55.0 (42.0-60.0); *n* = 23	55.0 (45.0-57.0); *n* = 23	56.0 (49.0-59.3); *n* = 22	0.80
LVEDV (mL)	98.0 (73.0-127.0); *n* = 15	95.0 (81.5-119.0); *n* = 21	111.5 (93.5-138.0); *n* = 32	117.0 (72.5-139.0); *n* = 9	0.58	110.0 (68.5-133.0); *n* = 17	109.5 (86.8-132.3); *n* = 16	101.0 (85.5-142.0); *n* = 17	101.0 (84.0-123.5); *n* = 21	0.82
LVESV (mL)	40.0 (28.0-60.0); *n* = 15	42.0 (34.5-53.5); *n* = 21	49.5 (42.0-65.0); *n* = 32	65.0 (33.0-89.0); *n* = 9	0.23	48.0 (28.0-71.5); *n* = 17	47.0 (35.8-75.8); *n* = 16	45.0 (32.5-82.5); *n* = 17	45.0 (32.5-62.0); *n* = 21	0.71
LVH^∗∗^	14.3%; 2/14	55.0%; 11/20	43.6%; 17/39	60.0%; 6/10	**0.02**	35.3%; 6/17	42.1%; 8/19	47.6%; 10/21	42.1%; 8/19	0.45
E/E′ med	10.8 (7.8-12.6); *n* = 18	10.7 (9.0-15.0); *n* = 23	10.0 (8.2-12.1); *n* = 41	9.1 (8.0-14.1); *n* = 10	0.22	12.0 (9.7-13.5); *n* = 20	9.0 (7.8-10.7); *n* = 22	10.0 (8.0-11.7); *n* = 23	10.9 (8.4-15.3); *n* = 22	**0.03**
E/E′ lat	8.5 (6.6-8.5); *n* = 18	9.0 (7.2-11.6); *n* = 23	7.6 (6.3-10.0); *n* = 41	8.4 (6.7-13.5); *n* = 10	0.33	8.9 (7.0-10.5); *n* = 20	7.3 (6.1-10.1); *n* = 22	8.5 (7.0-10.0); *n* = 23	9.1 (6.8-12.8); *n* = 22	0.13

Bold values indicate *p* values < 0.05. ^∗^*p* values derived from statistical tests of equal means (or proportions) across quartiles using analysis of variance for continuous variables and the chi-square test for categorical characteristics. ^∗∗^LVH was based on left ventricular mass index (LVMI): LVMI > 95 g/m^2^ for female, LVMI > 115 g/m^2^ for male. E/E′ med: early mitral inflow divided by medial early diastolic mitral annular velocity; E/E′ lat: early mitral inflow divided by lateral early diastolic mitral annular velocity; Gal-3: galectin-3; HF: heart failure; LVEDV: left ventricular end-diastolic volume; LVESV: left ventricular end-systolic volume; LVH: left ventricular hypertrophy; LVEF: left ventricular ejection fraction; sST2: soluble interleukin-1 receptor-like 1.

**Table 3:** Predictors of the primary endpoint in univariate and multivariate analyses.

Variable	Univariate analysis	Multivariate analysis		
	*p* value	HR	95% CI	*p* value
Age (years)	**0.01**	1.11	1.03–1.19	**0.01**
Gal-3 (per 1 ng/mL)	**0.001**	1.61	1.07-2.42	**0.02**
sST2 (per 1 ng/mL)	**0.04**	1.01	0.94–1.08	**0.87**
Baseline LVEF (%)	**<0.001**	0.80	0.70–0.90	**<0.001**
NT-proBNP (per 100 ng/L)	**0.02**	0.99	0.95-1.01	0.33

Bold values indicate *p* values < 0.05. CI: confidence interval; Gal-3: galectin-3; HR: hazard ratio; LVEF: left ventricular ejection fraction; NT-proBNP: N-terminal pro-B-type natriuretic peptide; sST2: soluble interleukin-1 receptor-like 1.

**Table 4:** Biomarker concentrations stratified to left ventricular ejection fraction after one year.

Variable	LVEF < 50% (*n* = 29)	LVEF ≥ 50% (*n* = 67)	*p* value
*Baseline biomarkers*			
Gal-3 (ng/mL)	7.1 (6.2-9.1); *n* = 29	7.1 (5.5-8.4); *n* = 67	0.34
sST2 (ng/mL)	24.5 (19.0-35.4); *n* = 29	24.0 (18.9-29.8); *n* = 67	0.37
hs-CRP peak (mg/dL)	6.3 (2.3-20.3); *n* = 28	2.7 (1.3-6.9); *n* = 64	**0.01**
Troponin I peak (ng/L)	69.7 (22.8-160.2); *n* = 28	14.4 (1.8-49.4); *n* = 64	**<0.001**
CK-MB peak (U/L)	135.0 (53.2-339.6); *n* = 29	46.5 (10.7-141.0); *n* = 66	**0.01**
NT-proBNP peak (pg/mL)	1921.5 (855.5-4262.0); *n* = 22	812.0 (287.5-1874.8); *n* = 48	**0.01**

*Biomarkers at follow-up*			
Gal-3 (ng/mL)	9.3 (6.8-10.7); *n* = 26	7.8 (6.1-10.0); *n* = 63	0.17
sST2 (ng/mL)	30.3 (24.9-34.9); *n* = 26	30.1 (24.4-36.7); *n* = 63	0.45

*Changes* ^∗^ *of biomarker concentrations*			
Change in Gal-3 (ng/mL)	1.7 (-0.7-3.5); *n* = 26	0.6 (-0.6-2.8); *n* = 63	0.24
Change in sST2 (ng/mL)	3.9 (-5.5-11.8); *n* = 26	5.4 (-2.7-13.2); *n* = 63	0.73

Bold values indicate *p* values < 0.05. ^∗^Changes were calculated as the one-year level minus the baseline level of each biomarker. CK-MB: creatine kinase-muscle/brain; Gal-3: galectin-3; hs-CRP: high-sensitivity C-reactive protein; LVEF: left ventricular ejection fraction; NT-proBNP: N-terminal pro-B-type natriuretic peptide; sST2: soluble interleukin-1 receptor-like 1.

## Data Availability

The raw data of the study can be provided upon request with maintenance of confidentiality, privacy, and anonymity of the research participants.

## Supplementary Materials

Supplementary Materials Table S1: correlation of Gal-3 and sST2 concentrations at baseline and after one-year follow-up and changes of their concentrations with echocardiographic parameters. Table S2: correlation of Gal-3 and sST2 concentrations at baseline and after one-year follow-up and changes of their concentrations with clinical parameters.
