# Supplementary material for: Association of Galectin-3 and Soluble ST2, and Their Changes, with Echocardiographic Parameters and Development of Heart Failure after ST-Segment Elevation Myocardial Infarction
Source: Dis Markers. 2019 Oct 10;2019:9529053. doi: 10.1155/2019/9529053 (PMC6811795; doi:10.1155/2019/9529053)
Supplement: Supplementary Materials — Table S1: correlation of Gal-3 and sST2 concentrations at baseline and after one-year follow-up and changes of their concentrations with echocardiographic parameters. Table S2: correlation of Gal-3 and sST2 concentrations at baseline and after one-year follow-up and changes of their concentrations with clinical parameters. [file 9529053.f1.docx]

**Table S1.** Correlation of Gal-3 and sST2 concentrations at baseline, after one-year follow-up and changes of their concentrations with echocardiographic parameters.

| **Variable** | **Baseline** | | | | **Changes*** | | | | **Follow-up** | | | |
| --- | --- | --- | --- | --- | --- | --- | --- | --- | --- | --- | --- | --- |
|  | **Gal-3** | | **sST2** | | **Gal-3** | | **sST2** | | **Gal-3** | | **sST2** | |
|  | **Rho** | **p-value** | **Rho** | **p-value** | **Rho** | **p-value** | **Rho** | **p-value** | **Rho** | **p-value** | **Rho** | **p-value** |
| **Baseline echocardiography** | | | | | | | | | | | | |
| LVEF, % | NS | | -0.18 | 0.053 | NS | | NS | | NS | | NS | |
| TAPSE, mm | NS | | NS | |  |  |  |  |  |  |  |  |
| LVEDD, mm |  |  |  |  |  |  |  |  |  |  |  |  |
| LVMI, g/m^2^ |  |  |  |  |  |  |  |  |  |  |  |  |
| E’med, cm/s |  |  |  |  |  |  |  |  |  |  |  |  |
| E/E’med |  |  |  |  |  |  |  |  |  |  |  |  |
| E’ lat, cm/s |  |  |  |  |  |  |  |  |  |  |  |  |
| E/E’ lat |  |  |  |  |  |  |  |  |  |  |  |  |
| LA dimension, mm |  |  |  |  |  |  |  |  |  |  |  |  |
| LVEDV, ml |  |  |  |  |  |  |  |  |  |  |  |  |
| LVESV, ml |  |  |  |  |  |  |  |  |  |  |  |  |
| **Follow-up echocardiography** | | | | | | | | | | | | |
| LVEF, % | NS | | -0.25 | **0.01** | NS | | 0.26 | **0.01** | NS | | NS | |
| TAPSE, mm | NS | | NS | | NS | | NS | |  |  |  |  |
| LVEDD, mm | NS | | 0.28 | **0.01** |  |  |  |  |  |  |  |  |
| LVMI, g/m^2^ | NS | | 0.35 | **<0.001** |  |  |  |  |  |  |  |  |
| E’med, cm/s | NS | | NS | |  |  |  |  |  |  |  |  |
| E/E’med |  |  |  |  |  |  |  |  |  |  |  |  |
| E’ lat, cm/s |  |  |  |  |  |  |  |  |  |  |  |  |
| E/E’ lat |  |  |  |  |  |  |  |  |  |  |  |  |
| LA dimension, mm |  |  |  |  |  |  |  |  |  |  |  |  |
| LVEDV, ml | -0.26 | **0.02** | NS | |  |  |  |  |  |  |  |  |
| LVESV, ml | NS | | 0.26 | **0.02** |  |  |  |  |  |  |  |  |

Bolded text indicates p-values <0.05.

* changes were calculated as the one-year level minus the baseline level of each biomarker

E’ med - medial early diastolic mitral annular velocity, E/E’ med. – early mitral inflow divided by medial early diastolic mitral annular velocity, E’ lat - lateral early diastolic mitral annular velocity, E/E’ lat – early mitral inflow divided by lateral early diastolic mitral annular velocity, Gal-3 – galectin-3, LA – left atrium, LVEDD – left ventricular end diastolic diameter, LVEDV - left ventricular end-diastolic volume, LVESV - left ventricular end-systolic volume, LVEF - left ventricular ejection fraction, LVMI - left ventricular mass index, NS - non-significant, TAPSE - tricuspid annular plane systolic excursion, sST2- soluble interleukin‐1 receptor‐like 1

**Table S2.** Correlation of Gal-3 and sST2 concentrations at baseline, after one-year follow-up and changes of their concentrations with clinical parameters.

| **Variable** | **Baseline** | | | | **Changes*** | | | | **Follow-up** | | | |
| --- | --- | --- | --- | --- | --- | --- | --- | --- | --- | --- | --- | --- |
|  | **Gal-3** | | **sST2** | | **Gal-3** | | **sST2** | | **Gal-3** | | **sST2** | |
|  | **Rho** | **p-value** | **Rho** | **p-value** | **Rho** | **p-value** | **Rho** | **p-value** | **Rho** | **p-value** | **Rho** | **p-value** |
| **Baseline characteristics** | | | | | | | | | | | | |
| Age, years | 0.38 | **<0.001** | NS | | NS | | NS | | 0.22 | **0.04** | NS | |
| BMI, kg/m^2^ | NS | | NS | |  |  |  |  | NS | | NS | |
| **Clinical status and laboratory findings on admission** | | | | | | | | | | | | |
| Killip class | 0.20 | **0.03** | NS | | NS | | NS | | NS | | 0.23 | **0.03** |
| Hemoglobin, g/dl | -0.26 | **0.004** | NS | |  |  |  |  | -0.22 | **0.04** | NS | |
| hs-CRP, mg/dL | 0.18 | 0.058 | 0.19 | **0.047** | NS | | NS | | NS | | NS | |
| Troponin I, ng/mL | NS | | NS | | NS | | -0.24 | **0.03** |  |  |  |  |
| NT-proBNP, pg/mL | 0.36 | **0.001** | 0.38 | **<0.001** |  |  | -0.27 | **0.03** | 0.29 | **0.02** | NS | |
| **Gal-3, ng/mL** | **_** | | 0.48 | **0.04** |  |  | **_** | | **_** | | **_** | |
| **sST-2, ng/mL** | 0.48 | **0.04** | **_** | |  |  | **_** | | **_** | | **_** | |
| eGFR, ml/min/1.73m^2^ | -0.30 | **0.001** | NS | |  |  | NS | | -0.24 | **0.03** | NS | |
| Serum sodium, mmol/L | NS | | -0.20 | **0.03** |  |  |  |  | NS | | NS | |
| Total cholesterol, mg/dL | -0.23 | **0.01** | -0.24 | **0.01** | 0.26 | **0.01** | 0.28 | **0.01** |  |  |  |  |
| LDL, mg/dL | -0.12 | 0.07 | -0.19 | 0.059 | 0.24 | **0.03** | 0.23 | **0.04** |  |  |  |  |
| **Clinical status and laboratory findings at discharge** | | | | | | | | | | | | |
| Hemoglobin, g/dl | -0.19 | 0.05 | NS | | NS | | NS | | NS | | NS | |
| Serum creatinine, mg/dl | NS | | 0.19 | 0.054 |  |  |  |  |  |  |  |  |
| eGFR, mL/min/1.73m^2^ | -0.36 | **<0.001** | -0.24 | **0.01** |  |  |  |  | -0.27 | **0.02** | NS | |
| **Outcomes** | | | | | | | | | | | | |
| Hospitalization length, days | 0.18 | 0.055 | 0.35 | **<0.001** | NS | | -0.29 | **0.01** | NS | | NS | |
| Time in ICCU, days | 0.35 | **<0.001** | 0.45 | **<0.001** |  |  | -0.21 | **0.046** | 0.28 | **0.01** | 0.31 | **0.003** |
| NYHA class after one year | NS | | 0.31 | **0.02** |  |  | NS | | NS | | NS | |

Bolded text indicates p-values <0.05.

* changes were calculated as the one-year level minus the baseline level of each biomarker

BMI – body mass index, eGFR – estimated glomerular filtration rate, Gal-3 – galectin-3, hs-CRP – high-sensitive C-reactive protein, ICCU – intensive cardiac care unit, LDL - low density lipoprotein, NT-proBNP - N-terminal pro-brain natriuretic peptide, NS – non-significant, NYHA – New York Heart Association, sST2- soluble interleukin‐1 receptor‐like 1
